# A Small RNA Encoded in the Rv2660c Locus of *Mycobacterium tuberculosis* Is Induced during Starvation and Infection

**DOI:** 10.1371/journal.pone.0080047

**Published:** 2013-12-12

**Authors:** Joanna Houghton, Teresa Cortes, Olga Schubert, Graham Rose, Angela Rodgers, Megan De Ste Croix, Rudolf Aebersold, Douglas B. Young, Kristine B. Arnvig

**Affiliations:** 1 Division of Mycobacterial Research, MRC National Institute for Medical Research, London, United Kingdom; 2 ETH Zurich, Institute of Molecular Systems Biology, Zurich, Switzerland; University of Padova, Medical School, Italy

## Abstract

Enhanced transcription of the Rv2660c locus in response to starvation of *Mycobacterium tuberculosis* H37Rv encouraged addition of the predicted Rv2660c protein to an improved vaccine formulation. Using strand-specific RNA sequencing, we show that the up-regulated transcript is in fact a small RNA encoded on the opposite strand to the annotated Rv2660c. The transcript originates within a prophage and is expressed only in strains that carry PhiRv2. The small RNA contains both host and phage sequences and provides a useful biomarker to monitor bacterial starvation during infection and/or non-replicating persistence. Using different approaches we do not find any evidence of Rv2660c at the level of mRNA or protein. Further efforts to understand the mechanism by which Rv2660c improves efficacy of the H56 vaccine are likely to provide insights into the pathology and immunology of tuberculosis.

## Introduction

The pathogenesis of *Mycobacterium tuberculosis* depends on the ability of the bacteria to adapt to a range of environmental conditions in the infected host, and characterisation of relevant *in vivo* phenotypes is crucial for the rational design of improved therapies and selection of antigens for use in vaccines and immunodiagnostics [1]. Inclusion of antigens that are preferentially expressed by bacterial populations that persist in a non-replicating state may enhance the ability of vaccines to prevent reactivation disease, and effective protection of non-human primates by the recently described H56 vaccine provides an encouraging proof-of-concept for this strategy [2], [3]. The H56 vaccine combines two dominant antigens expressed in exponential culture with a novel antigen, Rv2660c, originally identified on the basis of enhanced transcription in a starvation model of *M. tuberculosis* growth arrest [4]. In addition, strong induction of Rv2660c has been associated with hypoxia-induced non-replicating persistence and the enduring hypoxic response [5], [6]. The recent application of strand-specific RNA sequencing (RNAseq) has uncovered an extensive repertoire of non-coding RNA in *M. tuberculosis*, including 3′ and 5′ untranslated regions, antisense transcripts and intergenic small RNAs (sRNAs) [7]–[9]. One of the novel sRNAs detected by RNAseq – ncRv12659 (originally referred to as MTS2048) [7], [10] – overlaps with the locus annotated as encoding Rv2660c, and the aim of the present study was to characterise ncRv12659 and to determine its relationship to the Rv2660c vaccine antigen. Our findings strongly suggest that the starvation-induced transcriptional signal is due to increased expression of ncRv12659 arising from the plus strand and not, as previously thought increased expression of the hypothetical Rv2660c mRNA encoded on the minus strand.

## Results

### Transcriptional mapping of Rv2660c locus

**Figure 1:**
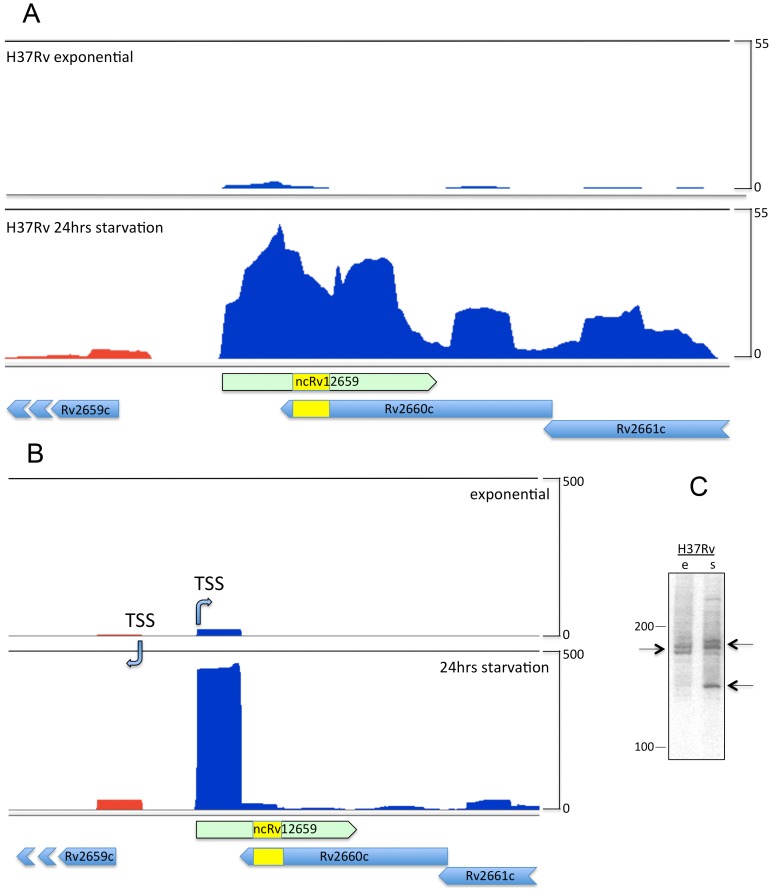
Mapping of ncRv12659 in *M. tuberculosis* H37Rv. A. Sequence analysis of total RNA in the Rv2660c locus. RNAseq data visualised with the Artemis genome browser [13] show mapping of reads from exponential (upper panel) and 24-hour starved cultures (lower panel). Reads mapping to the forward strand are shown in blue, and to the reverse strand in red. The *valU* insertion representing the boundary between PhiRv2 and host sequences is marked in yellow. The profile is dominated by ncRv12659, a forward strand transcript originating within PhiRv2. A proportion of reads continue beyond the 3′ end of ncRv12659, as determined by 3′ RACE. Reads have been normalised to total number of reads and adjusted to the same scale. B. Transcription start site (TSS) mapping. The forward ncRv12659 TSS maps to a location 62 nucleotides downstream of the reverse strand TSS for Rv2659c (PhiRv2 integrase). Promoter activity assessed by normalised read counts increases at both TSSs in response to starvation (lower panel). C. Northern blot analysis of ncRv21659. Expression of ncRv12659 in extracts from exponential (e) and stationary (s) phase cultures of *M. tuberculosis* H37Rv. 20 µg of total RNA was separated on a denaturing gel, transferred to a membrane and hybridised to a probe specific for the 3′ end of ncRv12659. The blot shows two transcripts of approximately 175 nucleotides in size from exponential phase RNA (indicated by arrows), corresponding to the size determined by RACE (see text). In stationary phase a slightly larger transcript as well as a smaller of around 125 nucleotides is also visible (arrow).

The Rv2660c mRNA was originally described as the most highly up-regulated transcript according to microarray analysis of *M. tuberculosis* H37Rv starved for 24 or 96 hours in PBS [4]. It was annotated as a questionable open reading frame located adjacent to the PhiRv2 prophage, which is integrated into the *valU* tRNA gene in many strains of *M. tuberculosis* [11], [12]. Several of the phage genes, including Rv2659c encoding the phage integrase, were also up-regulated in the microarray study [4]. Recently we identified a number of sRNAs including MTS2048/ncRv12659, which is convergent and overlapping with the Rv2660c open reading frame [7]. In order to investigate the relationship between the two genes further we compared transcriptomes from *M. tuberculosis* H37Rv from exponential growth and after 24 hours of starvation in PBS. Figure 1 illustrates RNAseq profiling (visualized in the Artemis Genome Browser, [13]) of the two transcriptomes and confirms the prominent induction of the Rv2660c locus but demonstrates that transcription occurs on the forward rather than the reverse strand (Figure 1A). In agreement with the recent annotation of non-coding RNA in *M. tuberculosis* we refer to this transcript as ncRv12659 [10]. In order to precisely determine the termini of the ncRv12659 transcript we mapped the 5′ and 3′ ends by RACE. A transcription start site (TSS) located at position 2980911, between the TSS of the PhiRv2 integrase (Rv2659c) and the right hand phage boundary marked by the *attR* duplication, was identified by 5′ RLM-RACE [14] of ncRv12659. An identical start site was found by RNAseq-based TSS mapping [15], with a marked increase in peak height in starved cultures (Figure 1B) [16]. The dominant 3′ end of ncRv12659 was identified by 3′ RACE to be at position 2981083, with additional 3′ ends detected at positions 2981010, 2981011, 2981026, 2981047, 2981055 and 2981077, but we did not identify any canonical intrinsic terminators (i.e. a stem-loop followed by a poly-U stretch) in this region. The total length of 173 nucleotides established by RACE is in good agreement with the largest prominent transcript identified by Northern blot (Figure 1C). The blot also revealed a series of smaller transcripts with a particularly prominent one around 120 nucleotides in stationary phase. Since we identified only one TSS and no apparent terminators for ncRv12659, we assume that the smaller transcripts and possibly also the main transcript are a result of post-transcriptional processing of a longer primary transcript.

**Figure 2:**
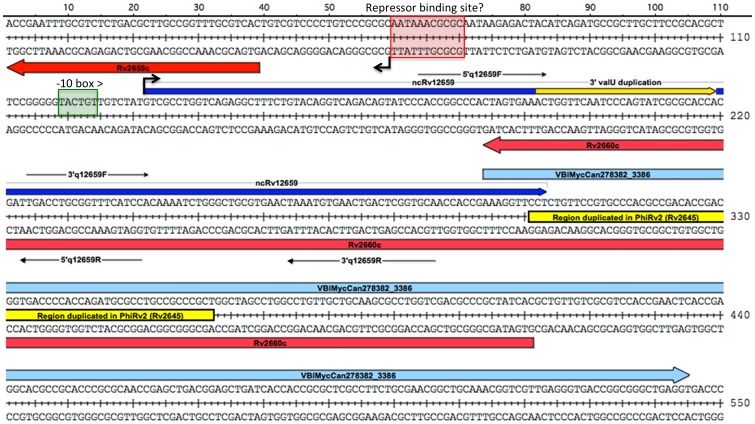
Details of region surrounding ncRv12659. The diagram shows the sequence around ncRv12659 (shown in blue) with relevant annotations mentioned in the text. Repeat regions are shown in yellow and open reading frames, for the hypothetical VBIMycCan278382_3386 on the plus strand and the hypothetical Rv2660c on the minus strand are shown in red. Black arrows indicate mapped TSSs and the predicted -10 box for the ncRv12659 promoter is shown in green; we did not identify a -10 box for Rv2659c. Red box indicates putative repressor binding site identified upstream of several PhiRv1 and PhiRv2 genes (see also Figures 5 and S2 and their legends). Location of primers used for qRT-PCR of ncRv12659 are indicated.

The ncRv12659 transcript comprises 60 nucleotides of PhiRv2 sequence followed by 28 nucleotides duplicated from the 3′ end of *valU* and a further 87 nucleotides of host sequence (Figure 2). The forward sequence after the sRNA contains a stretch of 61 nucleotides that have an almost exact duplication at the *attL* end of PhiRv2 annotated as part of Rv2645. Alternative annotations have been proposed for this region. For example in *Mycobacterium canettii* it is annotated as an open reading frame on the forward strand with homology to a partial protein sequence annotated in *Salmonella typhimurium* (VBIMycCan278382_3386). We did not detect transcripts on the reverse strand corresponding to the proposed Rv2660c and the upstream Rv2661c mRNAs; in both cases, the transcription profile is dominated by reads mapping to the opposite (i.e. plus) strand.

To screen for expression of Rv2660c protein we performed mass spectrometric analysis of protein extracts from exponential and starved cultures of *M. tuberculosis*. For this purpose we used the targeting approach of selected reaction monitoring (SRM), which is the method of choice for the sensitive detection of pre-determined low abundance proteins in complex samples by mass spectrometry [17]. Using SRM, more than 70% of annotated *M. tuberculosis* proteins, spanning the whole dynamic range of the proteome are detectable in unfractionated cell lysates from liquid cultures and the lower limit of detection has been estimated to be in general below 10 protein copies per cell [18], [19]. In the present study, during a starvation time course over 96 hours we were unable to detect any of the three mass spectrometry-compatible tryptic peptides predicted for Rv2660c in spite of corresponding synthetic reference peptides being detectable (Figure S1).

Combined transcript and protein data from exponential and starved cultures lead us to conclude that the dramatic up-regulation observed in starved cultures of *M. tuberculosis* H37Rv reflects expression of an sRNA (ncRv12659) with little or no contribution from the putative Rv2660c mRNA.

### Expression of ncRv12659 in clinical isolates

Localisation of the TSS of ncRv12659 within PhiRv2 suggested that the sRNA should only be expressed in strains that carry the phage, and raised the possibility that in the absence of a competing antisense RNA we might detect Rv2660c expression in PhiRv2-negative strains. To test these hypotheses, we screened the RNAseq profiles of five clinical isolates [20] and of the PhiRv2-negative *Mycobacterium bovis* BCG vaccine strain. N0072 and N0153 belong to *M. tuberculosis* Lineage 1 and lack PhiRv2. N0031, N0052 and N0145 all belong to Lineage 2 and are PhiRv2-positive (Table 1).

**Table 1:** Strains used in this study.

Strain	lineage	PhiRv2	TSS	RNAseq	Northern	qRT-PCR
*M. tuberculosis* H37Rv	4	+	Y	Y	Y	Y
*M. tuberculosis* N0031	2	+	N	(Y)	N	N
*M. tuberculosis* N0052	2	+	N	Y	Y	N
*M. tuberculosis* N0072	1	−	N	(Y)	Y	Y
*M. tuberculosis* N0145	2	+	(Y)	Y	N	N
*M. tuberculosis* N0153	1	−	Y	Y	N	N
*M. bovis* BCG	6	−	N	(Y)	Y	N

Yes/No indicates, which methods were applied to each strain, brackets indicate that the experiment was performed but data not shown.

**Figure 3:**
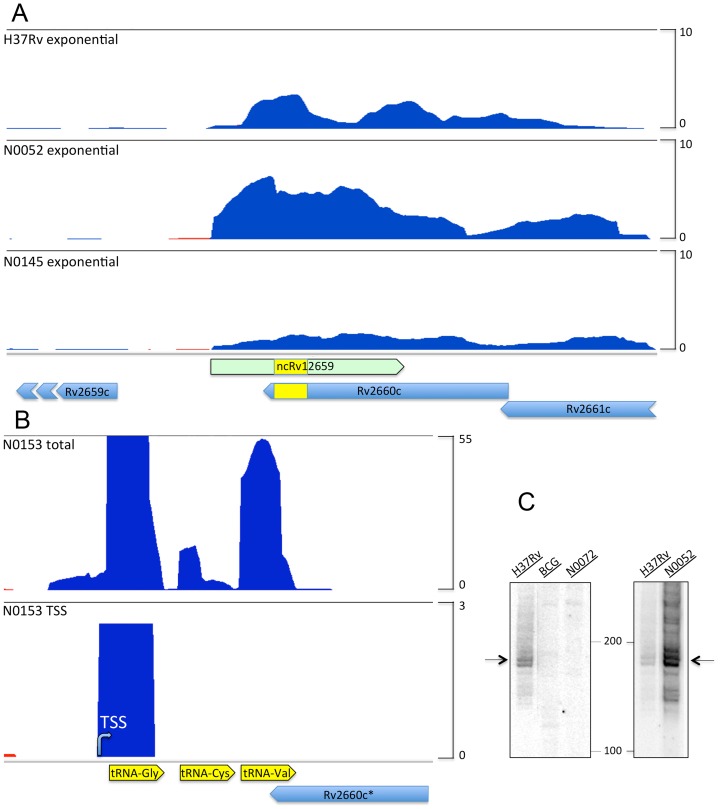
RNAseq profiling of the Rv2660c locus in *M. tuberculosis* clinical isolates. A. Lineage 2 isolates. RNaseq profiles of two PhiRv2-positive isolates compared to H37Rv, are dominated by the ncRv12659 forward transcript (blue) with no detectable reverse strand transcription of Rv2660c. Reads have been normalised to total number of reads and adjusted to same scale, and indicate higher expression of ncRv12659 in N0052 than in H37Rv and N0145. B. Lineage 1 isolate. RNAseq profile (top) and TSS mapping (bottom) of the PhiRv2-negative isolate N0153. Here the tRNA (Gly-Cys-Val) transcript overlaps with the hypothetical Rv2660c. Similar to PhiRv2-positive transcription profiles, the profile for the PhiRv2-negative N0153 is dominated by a forward transcript that in this case initiates at the tRNA promoter. There is no evidence of an Rv2660c mRNA even in the absence of ncRv12659. C. Northern blot analysis. Northern blots probed for ncRv12659, using 20 µg exponential phase RNA from the PhiRv2-positive *M. tuberculosis* H37Rv and N0052, and PhiRv2-negative *M. tuberculosis* N0072 and *M. bovis* BCG. The probe hybridised to the 3′ end of the ncRv12659 sequence to ensure compatibility with PhiRv2-negative strains; arrows indicate the position of ncRv12659 in the PhiRv2-positive strains and the absence of signal in the PhiRv2-negative strains.

RNAseq profiles of the two PhiRv2-positive N0052 and N0145, shown in Fig. 3A and clearly demonstrate similar patterns, although the levels of ncRv12569 differ slightly between strains, but more importantly no detectable signal from the reverse strand Rv2660c (Figure 3A). In addition, TSS mapping for *M. tuberculosis* N0145 identified the same primary transcript as in H37Rv, with a significant increase in stationary phase. The RNAseq profile of the PhiRv2-negative N0153 suggests that a minimal level of reads can be mapped to the region beyond the 3′ of the mature *valU* (Figure 3B). Northern blot analysis confirmed the presence of ncRv12659 only in PhiRv2-positive strains, and also confirmed the finding from the RNAseq data that ncRv12659 is more abundant in N0052 (Figure 3C). The blots revealed a faint signal of approximately 300 nucleotides specific for the PhiRv2-negative strains, which we presume corresponds to the 3′ end of the unprocessed tRNA transcript (Figure 3B). More importantly however, we found no evidence of a reverse strand Rv2660c mRNA in any of the strains regardless of whether ncRv12659 was expressed or not.

### Expression of ncRv12659 during infection

**Figure 4:**
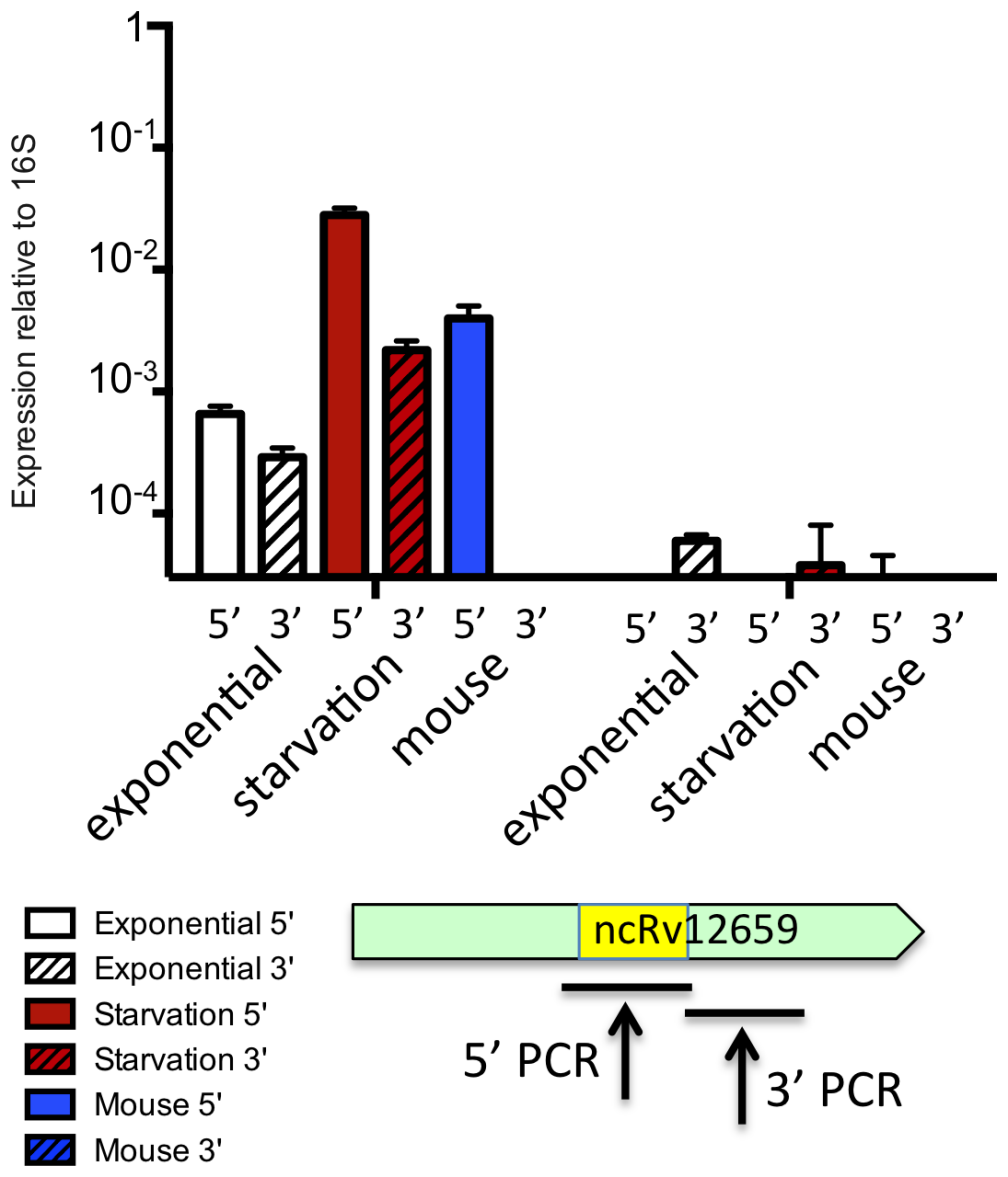
Expression of ncRv12659 during infection. Mycobacterial RNA extracted from tissues of mice infected with PhiRv2-positive *M. tuberculosis* H37Rv or PhiRv2-negative *M. tuberculosis* N0072 was compared to RNA from exponential and starved cultures of the same strains. The levels of ncRv12659 was measured by qRT-PCR using amplicons specific for the 5′ or 3′ regions of ncRv12659 and normalised to the level of 16S rRNA. ncRv12659 was induced during infection, though to a lower extent than during *in vitro* starvation; the 5′ portion of the sRNA accumulated to levels much higher than the 3′ end. The low level of 3′ amplicon detected in *in vitro* cultures of N0072 was not seen *in vivo*. Data represent mean and standard deviation of three biological replicates.

To characterise expression of ncRv12659 during intracellular growth we employed the mouse model used by Andersen and co-workers in development of the H56 vaccine [2]. Mice were infected with PhiRv2-positive *M. tuberculosis* H37Rv or PhiRv2-negative *M. tuberculosis* N0072 for 31 days, and expression of *M. tuberculosis* RNA recovered from tissues was measured by qRT-PCR. In order to distinguish expression of the PhiRv2-derived ncRv12659 RNA from the 3′ tRNA signal in the absence of PhiRv2, we used two amplicons. One (5′ amplicon) had a forward primer within the PhiRv2-derived sequence, and hence this amplicon is present only in H37Rv (and other PhiRv2 positive strains). The second (3′ amplicon) was located entirely downstream of the tRNA repeat and hence this amplicon represents an *M. tuberculosis* core sequence, present in both H37Rv and N0072 (Figure 4). The maximum value obtained for the 5′ amplicon in N0072 samples (3×10^−5^) was set as the baseline, since this region is not present in PhiRv2-negative strains and any 5′ signal from PhiRv2-negative strains should therefore be considered as noise. This method confirmed a 50-fold starvation-induced increase in RNA levels for the 5′ amplicon in *M. tuberculosis* H37Rv compared to exponential phase levels, and a six-fold increase during infection (Figure 4). The 3′ amplicon was detected at a level approximately two-fold lower than the 5′ amplicon in H37Rv during exponential growth, with a marked reduction in the relative level in starvation and decreasing below baseline during infection (Figure 4). Finally, we observed a very low level of the 3′ amplicon from N0072 exponential phase samples and even less after starvation, implying that transcriptional readthrough from the tRNA operon is minimal and only the PhiRv2-derived promoter is induced by starvation. Moreover, since our qRT-PCR was not strand specific, the results for the 3′ amplicon, which corresponds to the middle of the proposed Rv2660c sequence, also demonstrate that there is no detectable expression of Rv2660c mRNA in *M. tuberculosis* N0072 during mouse infection.

### Origin and function of ncRv12659

**Figure 5:**
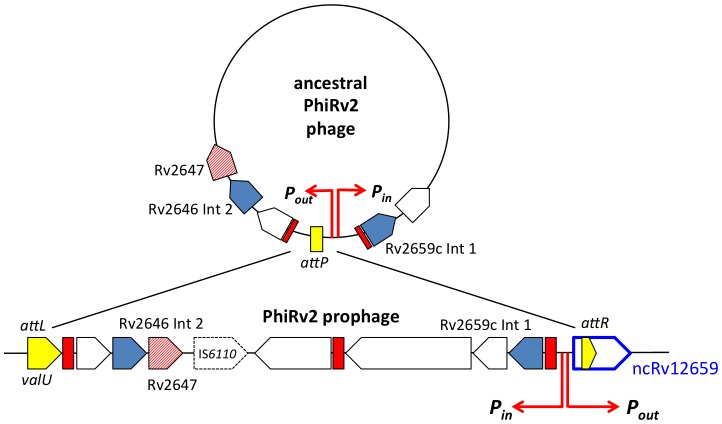
Hypothetical diagram of PhiRv2 as a circular virion. After integration into the *M. tuberculosis valU* tRNA gene the Rv2645/2646/2647 promoter from the PhiRv2 circular virion drives outward expression of the sRNA ncRv12659. 51 amino acids from PhiRv2 ORF Rv2647 show structural homology with the DNA binding domain of KorB repressor from plasmid RP4 [35]. A 14-nucleotide repeat sequence upstream of several phage genes represents a potential repressor binding site, shown in red.

Comparison with other temperate bacteriophages suggests that the original function of the ncRv12659 promoter was to drive expression of genes in the circular chromosome of the lytic phage (Figure 5). In the case of PhiRv2, this includes Rv2645 (a fusion of duplicated host sequence and a flap endonuclease-like domain), Rv2646 encoding a second integrase, and KorB-like repressor Rv2647. These genes are separated from the remaining inversely oriented PhiRv2 genes by an IS*6110* insertion. PhiRv1, a second prophage present in *M. tuberculosis* H37Rv, has a similar organisation, with a single integrase and two repressor-like proteins; again with inward and outward promoters at the right hand end of the integrated lysogen (Figure S2). The presence of host as well as phage sequences in the sRNA transcript driven by activation of the ncRv12659 promoter, and the fact that the sequence is highly conserved in the lysogen suggests that it could have a functional impact on the physiology of *M. tuberculosis*. To explore this possibility we made a construct in which expression of ncRv12659 was driven by its native promoter on a replicating plasmid (pMSC12659). The construct contains 131 basepairs upstream of the ncRv12659 TSS, including the promoter and the first 13 codons of Rv2659c as well as 170 basepairs downstream of the mapped 3′ end of ncRv12659 to ensure that any putative intrinsic termination signals were included (basepairs 1–471 in Figure 2).

**Figure 6:**
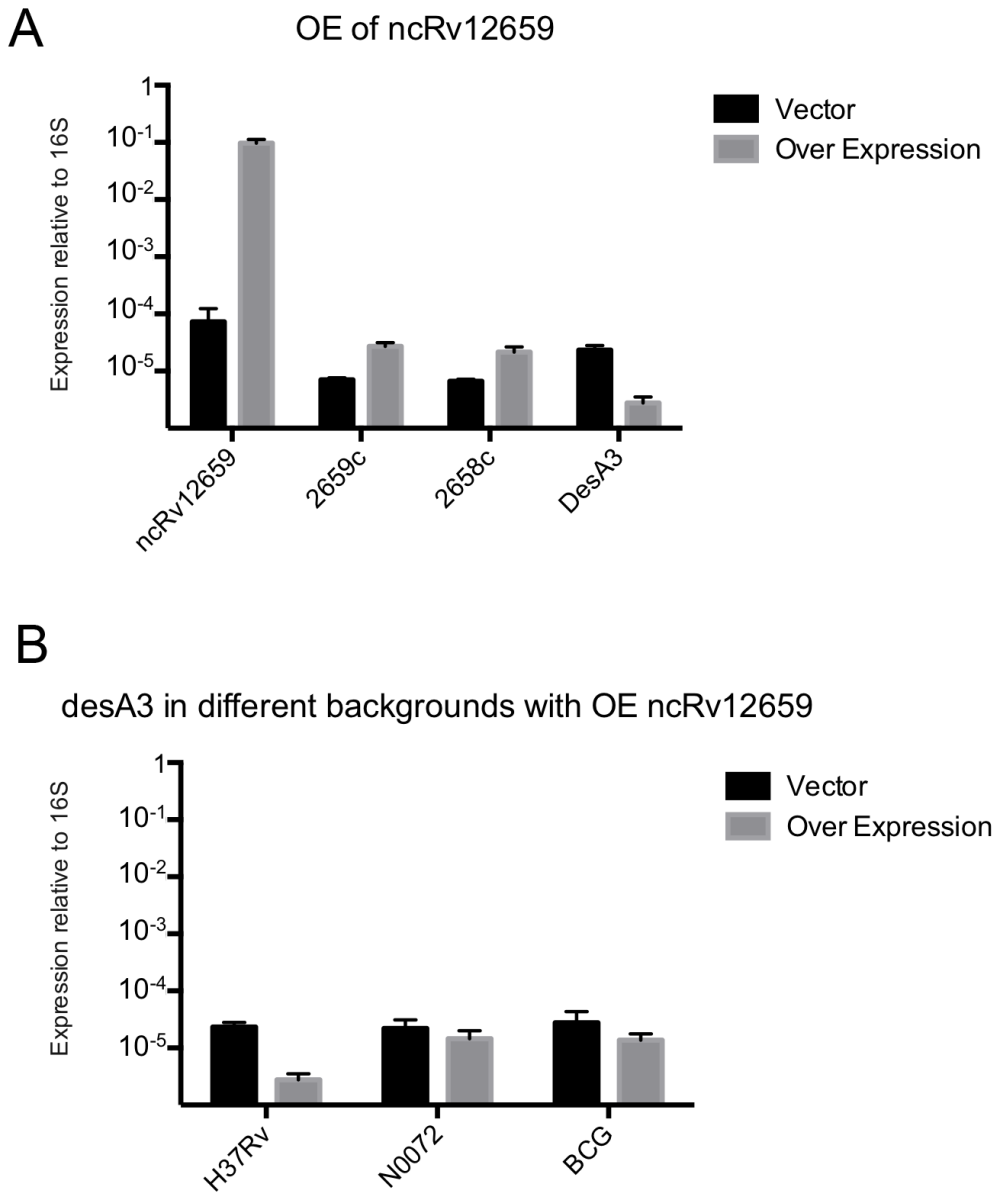
Expression analysis by qRT-PCR. Panel A shows the levels of ncRv12659, Rv2659c, Rv2658c and desA3 in the overexpression strain compared to strain with empty vector control. Transcript levels were measured by qRT-PCR and normalised to 16S rRNA levels. Results represent mean and standard deviation of three biological replicates. Panel B shows the expression levels of desA3 (Rv3229c) in three different backgrounds overexpressing ncRv12659. Transcript levels were measured by qRT-PCR and normalised to 16S rRNA levels. Results represent mean and standard deviation of three biological replicates.

The plasmid was transformed into PhiRv2-positive *M. tuberculosis* H37Rv as well as the PhiRv2-negative *M. tuberculosis* N0072. Analysis by qRT-PCR revealed that expression of ncRv12659 in *M. tuberculosis* H37Rv increased ∼1000-fold reaching approximately 10% of 16S rRNA levels, and furthermore that the level of ncRv12659 expression was similar in both strains. (Figure S3). The overexpression of ncRv12659 resulted in a small but detectable reduction in the growth rate of both strains (Figure S4). Microarray analysis of H37Rv ± pMSC12659 was performed using *M. tuberculosis* gene expression arrays from Agilent Technologies. Using a minimum fold-change of 2 and a p-value<0.05 we observed differential expression of more than 100 probes, corresponding to 68 phage as well as host genes (Table S1a). We decided to focus on probes with a minimum fold change of four, which left 27 probes (Table S1b). Six of these probes corresponded to sequences contained in the overexpression construct and were therefore disregarded, leaving 21 probes representing 11 genes (Table 2). Thirteen of the probes (fold change 6–113) mapped to a set of genomic loci containing partial duplications of a common core sequence (Figure S5). The repeat loci have been linked to diverse hypothetical protein products in different genome annotations, though the frameshifts present in the nucleotide alignment suggest that coding capacity is not conserved between the different copies. Five probes associated with PhiRv2 indicated a 4 to 5-fold up-regulation of Rv2659c and Rv2658c, and these changes were verified by qRT-PCR to be 3.9 and 3.2, respectively (Figure 6A). In spite of the reduced growth rate, only one gene, desA3 (Rv3229c) represented by three probes, was down-regulated more than the four-fold cut-off, with a probe average of 7-fold; an 8.2-fold down-regulation was verified by qRT-PCR (Figure 6A). A parallel analysis of *desA3* expression in *M. tuberculosis* N0072 ± pMSC12659 and *M. bovis* BCG ± pMSC12659 revealed only a slight reduction (1.5-fold and 2-fold respectively), suggesting that the more pronounced effect in H37Rv may be influenced by the presence of PhiRv2 genes other than ncRv12659 (Figure 6B).

**Table 2:** Differential gene expression upon overexpression of ncRv12659.

GeneName	FC OE/wt	Av. Of probes	Regulation	Repeat locus
MT1650.1	113.3	113	up	4
MT1560.1	80.5	80	up	3
MT0270.2	50.3	50	up	1
Rv1137c	31.8	32	up	2
Rv3613c	8.3	8	up	6
MT2423.1	95.8	6	up	5
MT2423.1	81.5	-	-	-
MT2423.1	62.6	-	-	-
Rv0257	90.4	6	up	1
Rv0257	86.2	-	-	-
Rv0257	21.9	-	-	-
Rv3612c	5.8	6	up	6
Rv3612c	5.7	-	-	-
Rv2659c	4.5	4	up	n/a
Rv2659c	3.8	-	-	-
Rv2658c	4.6	4	up	n/a
Rv2658c	3.8	-	-	-
Rv2658c	3.6	-	-	-
Rv3229c	4	7	down	n/a
Rv3229c	8	-	-	-
Rv3229c	9	-	-	-

FC shows values for individual probes (see Table S1), repeat loci see Figure S5.

## Discussion

We have demonstrated by RNAseq and Northern blot that the starvation-induced transcriptional signal ascribed to Rv2660c mRNA is in fact associated with an sRNA encoded on the opposite strand of the DNA rather than the mRNA. A similar profile was recently reported by Sala and co-workers [9]. Examination of a panel of *M. tuberculosis* clinical isolates under a variety of culture conditions failed to show any evidence of an Rv2660c mRNA, and we were unable to detect Rv2660c peptides by highly sensitive targeted mass spectrometry.

Rosenkrands and co-workers investigated the *M. tuberculosis* proteome during long-term starvation and failed to identify Rv2660c [21], and neither Kelkar et al. nor Schubert et al. identified Rv2660c in their recent high coverage proteome mapping data [19], [22]. Zheng et al. did report Rv2660c identification in *M. bovis* BCG but with only two out of tens of thousands of mass spectra [23]. Hence, it remains questionable if the Rv2660c protein exists.

The sequence of the proposed hypothetical Rv2660c protein has several unusual features. Most of the sequence is present in all strains of *M. tuberculosis* and in the precursor-like mycobacterial strains represented by *M. canettii* [24], but lacks primary sequence or structural homology with any annotated proteins outside of this group. The C-terminal region of Rv2660c corresponds to antisense translation of the 3′ portion of a valine tRNA molecule. In PhiRv2-positive strains, part of the Rv2660c nucleotide sequence has an imperfect duplication within the bacteriophage protein Rv2645. In some genome annotations the N-terminal region of Rv2660c is extended to include part of the Rv2661c sequence; other annotations include an alternative open reading frame on the opposite strand.

The starvation-induced sRNA transcript, ncRv12659, originates within the PhiRv2 prophage, and ncRv12659 is found only in strains that have retained PhiRv2. It is likely that PhiRv2 infected an early ancestor of the *M. tuberculosis* complex, but has undergone progressive erosion with loss of tail genes and deletion from many extant clinical isolates. Our results are consistent with a model in which the ncRv12659 promoter would have had a primary role in gene expression in the free circular virion and is relatively repressed in the stable lysogen. Activation of the ncRv12659 promoter together with the Rv2659c integrase promoter in response to starvation may reflect the release of repression as part of a programme to initiate the lytic cycle of the phage. The decision between lysis and lysogeny has previously been shown to be tightly linked to the nutrient availability of the host in many phages e.g. [25]–[27], and may explain why the ncRv12659 promoter responds so dramatically to starvation.

As described for other sRNAs [7], [10], ncRv12659 accumulates to high levels during *M. tuberculosis* infection and provides a potential biomarker for detection of cells that are starved for nutrients as well as oxygen, and which may represent future persisters [4]–[6]; in this case specifically for PhiRv2-positive strains. The observation that only the 5′ portion of ncRv12659 is detected during infection is intriguing and suggests premature termination of transcription or internal processing followed by degradation of a less stable 3′ portion of the transcript. It may be that the inclusion of part of the tRNA has a stabilising effect on the RNA.

The presence of host as well as phage sequences raised the possibility that expression of the sRNA transcript could affect the physiology of *M. tuberculosis*, and overexpression of ncRv12659 did result in impaired growth as well as perturbations in the transcription profile of *M. tuberculosis* H37Rv. More than fifty genes showed significant changes, the most highly up-regulated genes being PhiRv2 genes and a novel repeat locus. The observed induction of Rv2659c and massive increase in ncRv12659 level after transformation with pMSC12659 could result from sequestration of a repressor protein by provision of multiple copies of its binding site. We did not identify significant sequence similarities in the regions upstream of the TSSs of the up-regulated repeat loci. The mechanism by which over-expression of ncRv12659 leads to enhanced transcription of these sequences, and their potential coding (or non-coding) function remains to be determined. We conclude that the 5′ end of ncRv12659 provides a useful marker for phenotypic analysis of *M. tuberculosis* during infection with PhiRv2-positive strains, but that the distinctive transcriptional up-regulation of this locus in the starvation model is unlikely to play a direct role in the enhanced efficacy of the H56 vaccine against reactivation disease.

If Rv2660c represents a misannotation, how can we account for the beneficial effect of its addition to the H56 vaccine? There may be a fortuitous cross-reaction between the T cell response elicited by the recombinant Rv2660c protein and some as yet undefined antigen of *M. tuberculosis*. This would be consistent with the observed detection of T cell responses to Rv2660c in a panel of infected individuals [28]. Alternatively, but somewhat unlikely, there may be some highly specific environmental but yet unidentified condition that the bacteria encounter *in vivo* under which expression of Rv2660c is in fact induced. In light of the successful H56 vaccination data, identification of such a condition would provide an important insight into the physiology of latent infection. Finally, it's possible that fusion of the Rv2660c peptide to the remaining vaccine constituents has some beneficial effect on the way in which are processed and presented to T cells. Further efforts to understand the mechanism by which Rv2660c improves efficacy of the H56 vaccine are likely to provide insights into the pathology and immunology of tuberculosis and possibly also provide a further understanding of why a promising vaccine candidate such as MV85A has failed [29], [30].

## Materials and Methods

### Bacterial strains and plasmids

*E. coli* DH5α was used for plasmid constructions and grown in LB broth or on LB agar. Mycobacterial strains included the vaccine strain *Mycobacterium bovis* BCG, the laboratory strain *Mycobacterium tuberculosis* H37Rv and the clinical isolates N0031, N0052, N0072, N0145, N0153 [31].

### Growth of M. tuberculosis and M. bovis BCG

Cultures were grown in the standard Middlebrook 7H9 medium supplemented with glycerol (0.5%), Middlebrook ADC (10%) and Tween-80 (0.05%) in roller bottles at 2 rpm. Where required, kanamycin was added at 20 µg/ml. Exponential phase cultures were harvested at an OD_600_ 0.6 to 0.8; stationary phase cultures were harvested one week after OD_600_ had reached 1.0.

### Plasmid construction

The overexpression plasmid pMSC12659 was made by replacing an *Xba*I-*Hind*III promoter fragment of pKA303 [7] with a 483 basepair *Xba*I-*Hind*III fragment of the region around ncRv12659 including 131 basepairs upstream of the ncRv12659 TSS (Figure 2).

### RNA isolation

RNA isolation was done as described previously [32]. Briefly, cultures were harvested with rapid cooling by the addition of ice and pelleted at 10,000 rpm for 10 minutes. RNA was then isolated from the pellet using the FastRNA Pro Blue Kit from MP Biomedicals following the manufacturer's instructions. To isolate RNA from bacteria grown in mice, the lung homogenates were spun at 13,000 rpm for 5 minutes to collect the bacteria. These were resuspended in 1 ml Trizol (Invitrogen) with 150 micron glass beads and the samples disrupted in a fast Prep (MPBio) at a setting of 6.0 for 40 secs. The RNA was extracted according to manufacturer's guidelines. RNA concentration was measured by nanodrop and RNA integrity measured by the 2100 Bioanalyzer using a Nano chip.

### Preparation of starvation samples for RNAseq and proteomics

*M. tuberculosis* H37Rv was grown in Middlebrook 7H9 supplemented with 0.4% glycerol, 0.085% NaCl, 0.5% BSA and 0.05% Tyloxapol in roller bottle culture (2 rpm at 37°C). For nutrient-starvation experiments, exponentially growing bacteria were harvested as previously described [33] but using PBS supplemented with 0.025% Tyloxapol. RNA and protein was isolated from triplicate PBS-washed and 24 hours starved cultures.

### Northern blotting

Northern blotting was performed as previously described [32] using RNA marker low (Abnova) and 20 µg of RNA for each sample. Membranes were incubated with a probe constructed from the oligo template 2048nrtLONG 5′-gacctgcggtttcatccacaaaatctgggctgcgtgaactaaatgtCCTGTCTC-3′ in Ultrahyb (Ambion) at 68°C.

### Quantitative RT-PCR

Total RNA was treated with Turbo DNase (Ambion) until DNA free. cDNA was synthesized using Superscript III (Invitrogen) and random hexamers. Primers were designed using the Applied Biosystems software Primer Express, and sequences are listed in Table S2. Each 20 µl qRT-PCR reaction, contained 1× SYBRgreen (Applied Biosystems), 900 nm each primer and 5 µl of template cDNA. Absolute quantitation was perfomed and all genes were normalised to 16S expression.

### Mice and ethics statement

Groups of 6–8 week old Balb/C mice were infected by low-dose aerosol exposure with H37Rv *M. tuberculosis* and the N72 strain of *M. tuberculosis* using a Glas-Col (Terre Haute, IN) aerosol generator calibrated to deliver approximately 100 bacteria into the lungs. Bacterial counts in the lungs (*n* = 5) at each time point of the study were determined by plating serial dilutions of individual lung homogenates on duplicate plates of Middlebrook 7H11 agar containing OADC enrichment. Colony-forming units were counted after 3–4 weeks incubation at 37°C. Balb/C mice were bred and housed under specific pathogen free conditions at the Medical Research Council, National Institute for Medical Research. Protocols for experiments were performed, under project license number 80/2236, in accordance with Home Office (United Kingdom) requirements and the Animal Scientific Procedures Act, 1986.

### Whole transcriptome RNA sequencing

Isolation of RNA was performed as described above. All RNA samples were treated with Turbo DNase free (Ambion) until residue DNA contamination removed. Concentration and quality control of RNA samples was measured by Nanodrop (ND-1000, Labtech) and Agilent RNA chip (2100 Bioanalyser). Construction of strand-specific cDNA libraries from 2–3 µg total RNA was generated using the Illumina directional mRNASeq protocol (Part # 15018460 Rev. A); to capture all RNA species polyA-tail and size selection was omitted. Single-end read sequencing was performed on Illumina Genome Analyser and HiSeq platforms, using a single flow cell lane per library.

### Transcription Start Site (TSS) RNA sequencing

Strand-specific cDNA libraries for TSS mapping were made by Vertis Technologies AG, Germany (http://www.vertisbiotech.com/). In order to enrich for 5′ ends of primary transcripts, RNA was fragmented with ultrasound (4 pulses of 30 s at 4°C), treated with polynucleotide kinase (PNK) and then incubated with Terminator exonuclease (TEX, Epicentre), which specifically degrades RNA species which carry a 5′ monophosphate. The exonuclease-resistant RNA species (primary transcripts with 5′ PPP) were used for the construction of strand-specific cDNA libraries suitable for Illumina sequencing.

### RNAseq mapping

Raw reads were first filtered to discard low quality reads. Poor quality read bases were trimmed using the SolexaQA package [34]; default parameters were used, trimming bases with confidences p>0.05, and removing reads<25 bases. Reference based assembly using the reference genome H37Rv [EMBL:AL123456] was performed with BWA. Full data sets and accession numbers for transcriptomes are described elsewhere [16], [20].

### TSS mapping

Custom perl scripts were written for TSS calling. Briefly, for detecting candidate TSS, the increment in reads from one genome position to the next consecutive base was calculated for all genomic positions, selecting all genomic positions with an increment significantly above the average background coverage as candidates. Automated annotation of the putative TSS detected according to genomic distribution was performed as described by [15]. Full data sets and accession numbers for TSS mappings are described elsewhere [16], [20].

### Microarray Analysis

Whole genome *M. tuberculosis* microarray slides were purchased from Agilent Technologies through the Bacterial Microarray Group at St. George's (BμG@S), University of London. For cDNA synthesis 2 µg of vector control and over expression RNA, isolated from exponential cultures at OD_600 nm_ of 0.6 was used. The cDNA was labelled individually with both Cy-3 and Cy-5 dyes (GE Healthcare) using Superscript III reverse transcriptase (Invitrogen). Dye swaps were performed and the cDNA hybridized to an 8 Chamber Agilent slide at 65°C for 16 hours before washing the slide with Oligo aCGH Wash Buffer 1 (Agilent) for 5 minutes at room temperature and Oligo aCGH Wash Buffer 2 (Agilent) for 1 minute at 37°C. Slides were stabilized using Agilent's Stabilisation and Drying Solution according to manufacturer's instructions.

Slides were scanned at 5 microns using an Agilent Technologies Microarray Scanner at BμG@S. Txt files created by the Agilent scanner were analysed using Genespring 12.0 filtering on flags and expression. T-test against zero was performed and p-value selected as p<0.05, correcting for multiple comparisons using Benjamini-Hochberg. The array design is available in BμG@Sbase (Accession No. A-BUGS-41; http://bugs.sgul.ac.uk/A-BUGS-41) and also ArrayExpress (Accession No. A-BUGS-41). Fully annotated microarray data have been deposited in BμG@Sbase (accession number E-BUGS-156; http://bugs.sgul.ac.uk/E-BUGS-156) and also ArrayExpress (accession number E-BUGS-156).

### Proteomics

Bacterial cell pellets were dissolved in lysis buffer containing 8 M urea and 0.1% RapiGest (Waters) in 0.1 M ammonium bicarbonate buffer. The cell suspension was thoroughly vortexed and incubated at room temperature for 10 min. Subsequently, cells were disrupted by ribolysing the samples at a setting of 6.5 for 30 secs at 4°C using 150 µm glass beads (SIGMA). Lysates were then centrifuged at 13,000 rpm for 5 minutes. The clarified lysate was filtered using 0.2 µm Millipore tubes (UFC30GV25). Protein concentration was determined using a BCA assay according to manufacturer's protocol (Thermo Fisher Scientific). Protein disulfide bonds were reduced by adding 5 mM tris(2-carboxyethyl)phosphine (TCEP) and incubating for 30 min at 37°C. Next, the free cysteine residues were alkylated by adding 10 mM iodoacetamide and incubating for 30 min in the dark at room temperature. Excessive iodoacteamide was captured by addition of 12.5 M N-acetyl cysteine and incubation for 10 min at room temperature. Extracted protein samples were diluted at a ratio of 1∶5 with 0.05 M ammonium bicarbonate buffer to reach a urea concentration of <2 M. Sequencing-grade modified trypsin (Promega) was added at a ratio of 1∶100 enzyme∶substrate (weight/weight) and incubated for over night at 37°C with gentle shaking at 300 rpm. To stop the tryptic digest and to precipitate RapiGest the pH was lowered to 2 using 50% trifluoro acetic acid (TFA) followed by an incubation for 30 min at 37°C with shaking at 500 rpm. Water-immiscible degradation products of RapiGest were pelleted by centrifugation at 16,000 g for 10 min. The cleared peptide solution was desalted with C18 reversed-phase columns (Waters). Prior to use, the C18 columns were activated with 100% acetonitrile (ACN), followed by equilibration with 2% ACN/0.1% TFA. After loading the sample, the columns were washed four times with 2% ACN/0.1% TFA. Finally, peptides were eluted with 50% ACN/0.1% TFA, dried under vacuum, and re-solubilised in 2% ACN/0.1% FA to a final concentration of 1.0 mg/ml.

For each of the 3 mass spectrometry-suitable tryptic peptides of Rv2660c a synthetic peptide was purchased in unpurified form (JPT Peptide Technologies), re-solubilised in 180 µl of 20% ACN/0.1% FA and spiked into the samples with a dilution of 1∶100 (v/v) as positive control. Additionally, 11 retention time peptides (Biognosys) were added to each sample. Peptides were separated by liquid chromatography on a fused silica microcapillary column (15 cm×75 µm) packed in-house with C18 resin (Magic C18 AQ 5 µm diameter, 200 Å pore size, Michrom BioResources) with a linear gradient from 98% solvent A (2% ACN/0.1% FA) and 2% solvent B (98% ACN/0.1% FA) to 35% solvent B over 35 min at a flow rate of 300 nl/min. Rv2660c peptides were measured in unscheduled selected reaction monitoring (SRM) acquisition mode on a 5500 QTRAP mass spectrometer (AB Sciex) equipped with a nanoelectrospray ion source. The optimal 5 SRM transitions per peptide precursor, as well as the chromatographic retention time, were obtained from the Mtb Proteome Library [19]. The mass spectrometer was operated in positive mode using electrospray ionisation. The SRM transitions were acquired with a mass window of 0.7 half-maximum peak width (unit resolution) in Q1 and Q3, a cycle time of <2 s and a dwell time of 20 ms. Collision energies were calculated as follows: CE = 0.044 * (m/z)+5.5 and CE = 0.051 * (m/z)+0.5 for 2+ and 3+ charged precursor ions, respectively. Data were analysed manually using the software Skyline. The SRM data can be viewed in and downloaded from Panorama: https://daily.panoramaweb.org/labkey/project/Aebersold/schubert/2013_Houghton_Rv2660c/begin.view?

## Supporting Information

Figure S1 **SRM analysis of tryptic peptides from Rv2660c.** SRM traces over 96 hours of a starvation experiment are shown for three tryptic peptides from Rv2660c and a peptide derived from Rv3457c serving as a positive control. The first column shows the SRM signals of the synthetic reference peptides spiked into the first time point. The other three columns show the SRM signals in samples without reference peptides spiked in. No signal for the targeted peptides can be detected, neither by zooming into the expected regions (not shown). The positive control peptide in the last row shows that the sensitivity as well as chromatographic retention times are highly reproducible within the different samples.
(TIF)

Figure S2 **PhiRv1 prophage.** The PhiRv1 genome has a structural organisation similar to PhiRv2, with adjacent inward and outward TSSs (shown as an Artemis trace). PhiRv1 encodes a single integrase (Rv1586c) and two predicted proteins with structural homology to transcriptional repressors (Rv1574, Rv1575) as well as a conserved putative repressor binding site, shown in red.
(TIF)

Figure S3 **Over-expression of ncRv12659.** The diagram shows the level of (over)expression of ncRv12659 measured by qRT-PCR and normalised to 16S levels in all three backgrounds used. Each bar represents the mean and standard deviation of three biological replicates.
(TIF)

Figure S4 **Growth of** ***M. tuberculosis*** **upon ncRv12659 overexpression.** The curves illustrate that both strains of *M. tuberculosis* had a significant growth defect when expressing high amounts of ncRv12659.
(TIF)

Figure S5 **Sequences of** ***M. tuberculosis*** **repeat loci.** Sequence alignments of the repeat loci with homology to Rv0257. The diagram illustrates the sequences of the individual repeats, their location and the genes or gene regions they are associated with. Cyan highlights probes mapping in forward orientation with respect to Rv0257 orientation and magenta highlights probes mapping in the antisense orientation.
(TIF)

Table S1 **Table shows Genespring output of probes with a minimum fold-change in expression of at least two, and their corresponding gene annotations** (A). T-test against zero was performed and p-value selected as p<0.05, correcting for multiple comparisons using Benjamini-Hochberg. (**B**) Table shows same data as Table S1 but with a cut-off value of four.
(XLSX)

Table S2 **Primers used for quantitative RT-PCR.**
(DOCX)

## References

[1] Barry CE 3rd, Boshoff HI, Dartois V, Dick T, Ehrt S, et al (2009) The spectrum of latent tuberculosis: rethinking the biology and intervention strategies. Nat Rev Microbiol 7: 845–855. 19855401 10.1038/nrmicro2236 PMC4144869

[2] Aagaard C, Hoang T, Dietrich J, Cardona PJ, Izzo A, et al (2011) A multistage tuberculosis vaccine that confers efficient protection before and after exposure. Nature medicine 17: 189–194. 10.1038/nm.2285 21258338

[3] Lin PL, Dietrich J, Tan E, Abalos RM, Burgos J, et al (2012) The multistage vaccine H56 boosts the effects of BCG to protect cynomolgus macaques against active tuberculosis and reactivation of latent Mycobacterium tuberculosis infection. J Clin Invest 122: 303–314. 22133873 10.1172/JCI46252 PMC3248283

[4] Betts JC, Lukey PT, Robb LC, McAdam RA, Duncan K (2002) Evaluation of a nutrient starvation model of Mycobacterium tuberculosis persistence by gene and protein expression profiling. Mol Microbiol 43: 717–731. 11929527 10.1046/j.1365-2958.2002.02779.x

[5] Rustad TR, Harrell MI, Liao R, Sherman DR (2008) The enduring hypoxic response of Mycobacterium tuberculosis. PLoS One 3: e1502. 18231589 10.1371/journal.pone.0001502 PMC2198943

[6] Voskuil MI, Visconti KC, Schoolnik GK (2004) Mycobacterium tuberculosis gene expression during adaptation to stationary phase and low-oxygen dormancy. Tuberculosis 84: 218–227. 15207491 10.1016/j.tube.2004.02.003

[7] Arnvig KB, Comas I, Thomson NR, Houghton J, Boshoff HI, et al (2011) Sequence-based analysis uncovers an abundance of non-coding RNA in the total transcriptome of Mycobacterium tuberculosis. PLoS Pathogens 7: e1002342. 22072964 10.1371/journal.ppat.1002342 PMC3207917

[8] Pellin D, Miotto P, Ambrosi A, Cirillo DM, Di Serio C (2012) A genome-wide identification analysis of small regulatory RNAs in Mycobacterium tuberculosis by RNA-Seq and conservation analysis. PLoS One 7: e32723. 22470422 10.1371/journal.pone.0032723 PMC3314655

[9] Uplekar S, Rougemont J, Cole ST, Sala C (2012) High-resolution transcriptome and genome-wide dynamics of RNA polymerase and NusA in Mycobacterium tuberculosis. Nucleic acids research 10.1093/nar/gks1260. 10.1093/nar/gks1260 PMC3553938 23222129

[10] Lamichhane G, Arnvig KB, McDonough KA (2013) Definition and annotation of (myco)bacterial non-coding RNA. Tuberculosis 93: 26–29. 23291152 10.1016/j.tube.2012.11.010

[11] Hendrix RW, Smith MC, Burns RN, Ford ME, Hatfull GF (1999) Evolutionary relationships among diverse bacteriophages and prophages: all the world's a phage. Proceedings of the National Academy of Sciences of the United States of America 96: 2192–2197. 10051617 10.1073/pnas.96.5.2192 PMC26759

[12] Williams KP (2002) Integration sites for genetic elements in prokaryotic tRNA and tmRNA genes: sublocation preference of integrase subfamilies. Nucleic acids research 30: 866–875. 11842097 10.1093/nar/30.4.866 PMC100330

[13] Carver T, Harris SR, Berriman M, Parkhill J, McQuillan JA (2012) Artemis: an integrated platform for visualization and analysis of high-throughput sequence-based experimental data. Bioinformatics 28: 464–469. 22199388 10.1093/bioinformatics/btr703 PMC3278759

[14] Argaman L, Hershberg R, Vogel J, Bejerano G, Wagner EG, et al (2001) Novel small RNA-encoding genes in the intergenic regions of Escherichia coli. Curr Biol 11: 941–950. 11448770 10.1016/s0960-9822(01)00270-6

[15] Sharma CM, Hoffmann S, Darfeuille F, Reignier J, Findeiss S, et al (2010) The primary transcriptome of the major human pathogen Helicobacter pylori. Nature 464: 250–255. 20164839 10.1038/nature08756

[16] Cortes T, Schubert OT, Rose G, Arnvig KB, Comas I, et al (2013) Genome-Wide Mapping of Transcriptional Start Sites Defines an Extensive Leaderless Transcriptome in Mycobacterium tuberculosis. Cell Reports in press. 10.1016/j.celrep.2013.10.031 PMC3898074 24268774

[17] Picotti P, Aebersold R (2012) Selected reaction monitoring-based proteomics: workflows, potential, pitfalls and future directions. Nature methods 9: 555–566. 22669653 10.1038/nmeth.2015

[18] Picotti P, Bodenmiller B, Mueller LN, Domon B, Aebersold R (2009) Full dynamic range proteome analysis of S. cerevisiae by targeted proteomics. Cell 138: 795–806. 19664813 10.1016/j.cell.2009.05.051 PMC2825542

[19] Schubert OT, Mouritsen J, Ludwig C, Rost HL, Rosenberger G, et al (2013) The Mtb Proteome Library: A Resource of Assays to Quantify the Complete Proteome of Mycobacterium tuberculosis. Cell Host Microbe 13: 602–612. 23684311 10.1016/j.chom.2013.04.008 PMC3766585

[20] Rose G, Cortes T, Comas I, Coscolla M, Gagneux S, et al (2013) Mapping of genotype-phenotype diversity amongst clinical isolates of Mycobacterium tuberculosis by sequence-based transcriptional profiling. Genome Biology and Evolution doi: 10.1093/gbe/evt138 10.1093/gbe/evt138 PMC3814196 24115728

[21] Albrethsen J, Agner J, Piersma SR, Hojrup P, Pham TV, et al (2013) Proteomic Profiling of Mycobacterium tuberculosis Identifies Nutrient-starvation-responsive Toxin-antitoxin Systems. Mol Cell Proteomics 12: 1180–1191. 23345537 10.1074/mcp.M112.018846 PMC3650330

[22] Kelkar DS, Kumar D, Kumar P, Balakrishnan L, Muthusamy B, et al (2011) Proteogenomic analysis of Mycobacterium tuberculosis by high resolution mass spectrometry. Mol Cell Proteomics 10: M111 011627. 10.1074/mcp.M111.011627 PMC3275902 21969609

[23] Zheng J, Liu L, Wei C, Leng W, Yang J, et al (2012) A comprehensive proteomic analysis of Mycobacterium bovis bacillus Calmette-Guerin using high resolution Fourier transform mass spectrometry. J Proteomics 77: 357–371. 23000594 10.1016/j.jprot.2012.09.010

[24] Supply P, Marceau M, Mangenot S, Roche D, Rouanet C, et al (2013) Genomic analysis of smooth tubercle bacilli provides insights into ancestry and pathoadaptation of Mycobacterium tuberculosis. Nat Genet 45: 172–179. 23291586 10.1038/ng.2517 PMC3856870

[25] Los M, Golec P, Los JM, Weglewska-Jurkiewicz A, Czyz A, et al (2007) Effective inhibition of lytic development of bacteriophages lambda, P1 and T4 by starvation of their host, Escherichia coli. BMC biotechnology 7: 13. 17324284 10.1186/1472-6750-7-13 PMC1820593

[26] Slominska M, Neubauer P, Wegrzyn G (1999) Regulation of bacteriophage lambda development by guanosine 5′-diphosphate-3′-diphosphate. Virology 262: 431–441. 10502521 10.1006/viro.1999.9907

[27] Williams MD, Fuchs JA, Flickinger MC (1991) Null mutation in the stringent starvation protein of Escherichia coli disrupts lytic development of bacteriophage P1. Gene 109: 21–30. 1721886 10.1016/0378-1119(91)90584-x

[28] Govender L, Abel B, Hughes EJ, Scriba TJ, Kagina BM, et al (2010) Higher human CD4 T cell response to novel Mycobacterium tuberculosis latency associated antigens Rv2660 and Rv2659 in latent infection compared with tuberculosis disease. Vaccine 29: 51–57. 20974305 10.1016/j.vaccine.2010.10.022 PMC3376751

[29] Tameris M, McShane H, McClain JB, Landry B, Lockhart S, et al (2013) Lessons learnt from the first efficacy trial of a new infant tuberculosis vaccine since BCG. Tuberculosis 93: 143–149. 23410889 10.1016/j.tube.2013.01.003 PMC3608032

[30] Tameris MD, Hatherill M, Landry BS, Scriba TJ, Snowden MA, et al (2013) Safety and efficacy of MVA85A, a new tuberculosis vaccine, in infants previously vaccinated with BCG: a randomised, placebo-controlled phase 2b trial. Lancet 381: 1021–1028. 23391465 10.1016/S0140-6736(13)60177-4 PMC5424647

[31] Comas I, Chakravartti J, Small PM, Galagan J, Niemann S, et al (2010) Human T cell epitopes of Mycobacterium tuberculosis are evolutionarily hyperconserved. Nat Genet 42: 498–503. 20495566 10.1038/ng.590 PMC2883744

[32] Arnvig KB, Young DB (2009) Identification of small RNAs in Mycobacterium tuberculosis. Mol Microbiol 73: 397–408. 19555452 10.1111/j.1365-2958.2009.06777.x PMC2764107

[33] Gengenbacher M, Rao SP, Pethe K, Dick T (2010) Nutrient-starved, non-replicating Mycobacterium tuberculosis requires respiration, ATP synthase and isocitrate lyase for maintenance of ATP homeostasis and viability. Microbiology 156: 81–87. 19797356 10.1099/mic.0.033084-0

[34] Cox MP, Peterson DA, Biggs PJ (2010) SolexaQA: At-a-glance quality assessment of Illumina second-generation sequencing data. BMC Bioinformatics 11: 485. 20875133 10.1186/1471-2105-11-485 PMC2956736

[35] Kelley LA, Sternberg MJ (2009) Protein structure prediction on the Web: a case study using the Phyre server. Nat Protoc 4: 363–371. 19247286 10.1038/nprot.2009.2

