# Supplementary figures and images for: A Small RNA Encoded in the Rv2660c Locus of Mycobacterium tuberculosis Is Induced during Starvation and Infection
Source: PLoS One. 2013 Dec 12;8(12):e80047. doi: 10.1371/journal.pone.0080047 (PMC3861185; doi:10.1371/journal.pone.0080047)

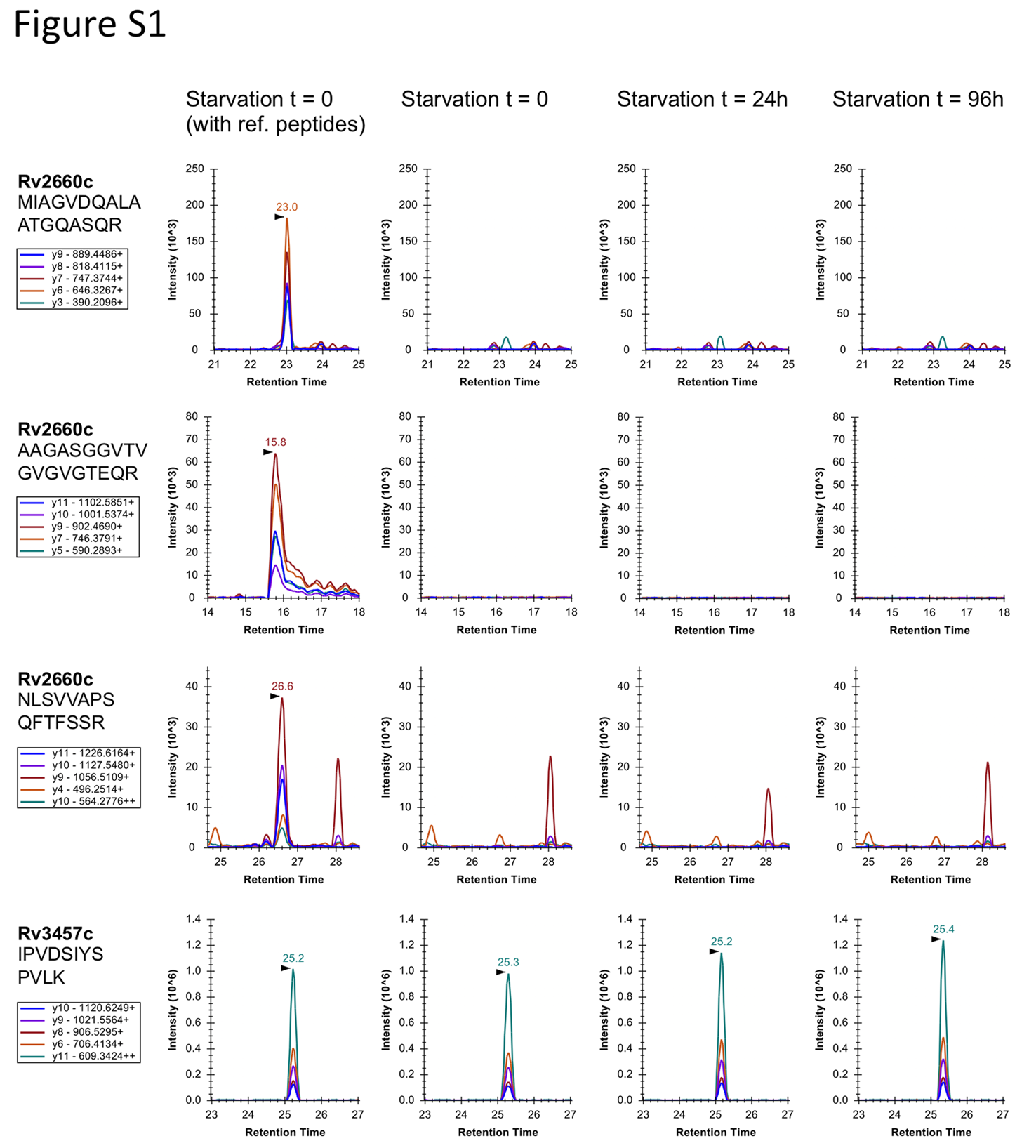

Supplement: Figure S1 — SRM analysis of tryptic peptides from Rv2660c. SRM traces over 96 hours of a starvation experiment are shown for three tryptic peptides from Rv2660c and a peptide derived from Rv3457c serving as a positive control. The first column shows the SRM signals of the synthetic reference peptides spiked into the first time point. The other three columns show the SRM signals in samples without reference peptides spiked in. No signal for the targeted peptides can be detected, neither by zooming into the expected regions (not shown). The positive control peptide in the last row shows that the sensitivity as well as chromatographic retention times are highly reproducible within the different samples. (TIF) [file pone.0080047.s001.tif]

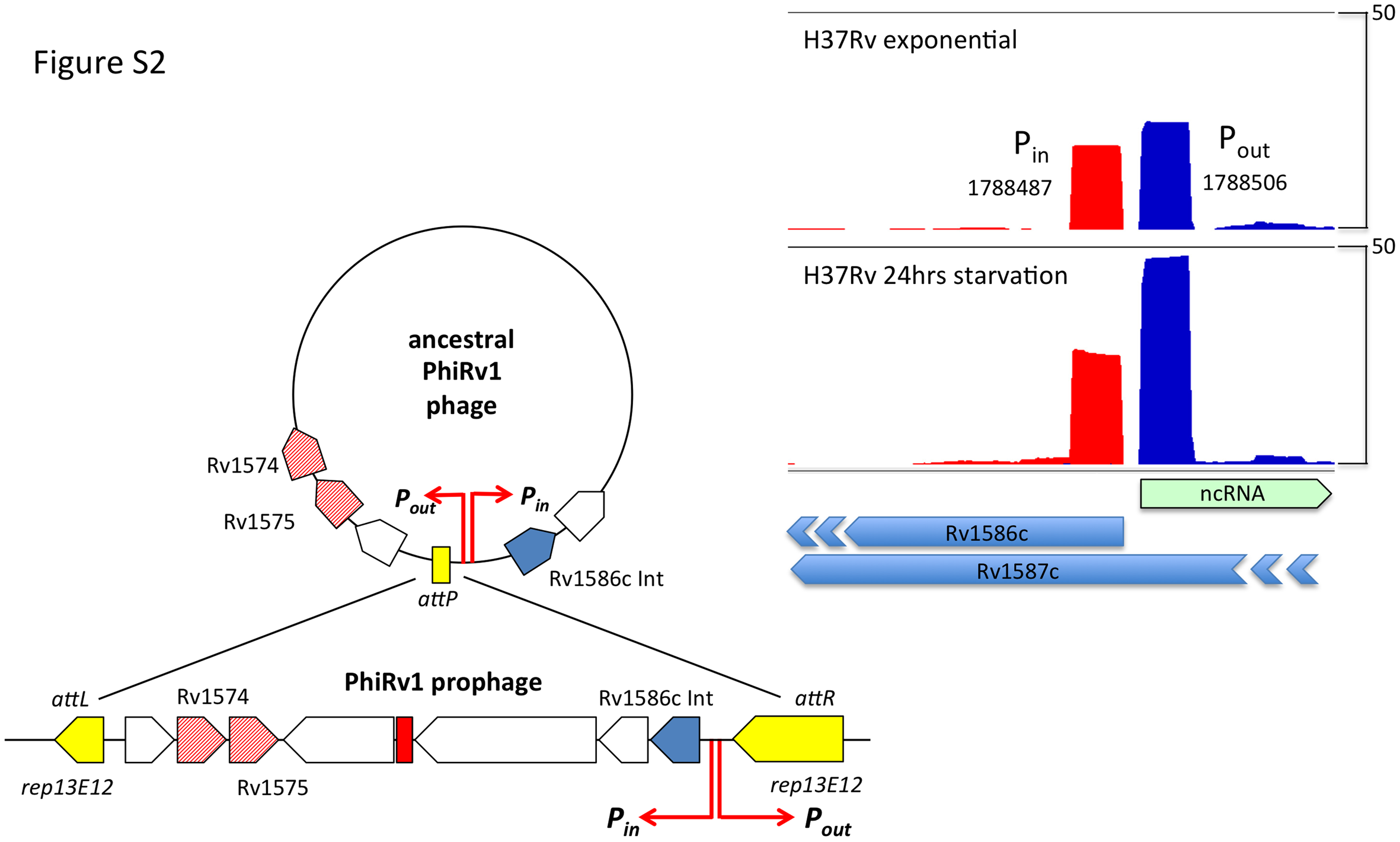

Supplement: Figure S2 — PhiRv1 prophage. The PhiRv1 genome has a structural organisation similar to PhiRv2, with adjacent inward and outward TSSs (shown as an Artemis trace). PhiRv1 encodes a single integrase (Rv1586c) and two predicted proteins with structural homology to transcriptional repressors (Rv1574, Rv1575) as well as a conserved putative repressor binding site, shown in red. (TIF) [file pone.0080047.s002.tif]

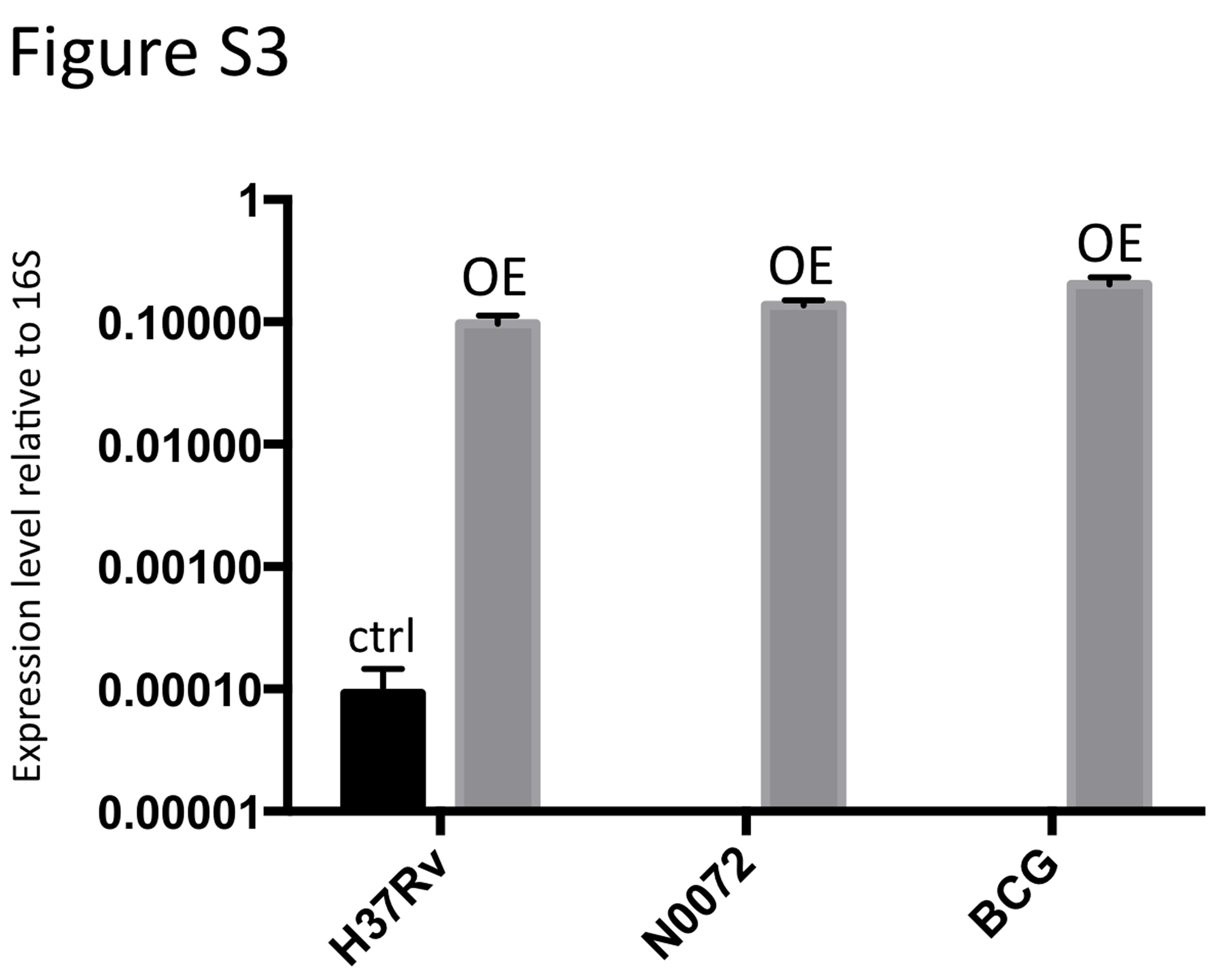

Supplement: Figure S3 — Over-expression of ncRv12659. The diagram shows the level of (over)expression of ncRv12659 measured by qRT-PCR and normalised to 16S levels in all three backgrounds used. Each bar represents the mean and standard deviation of three biological replicates. (TIF) [file pone.0080047.s003.tif]

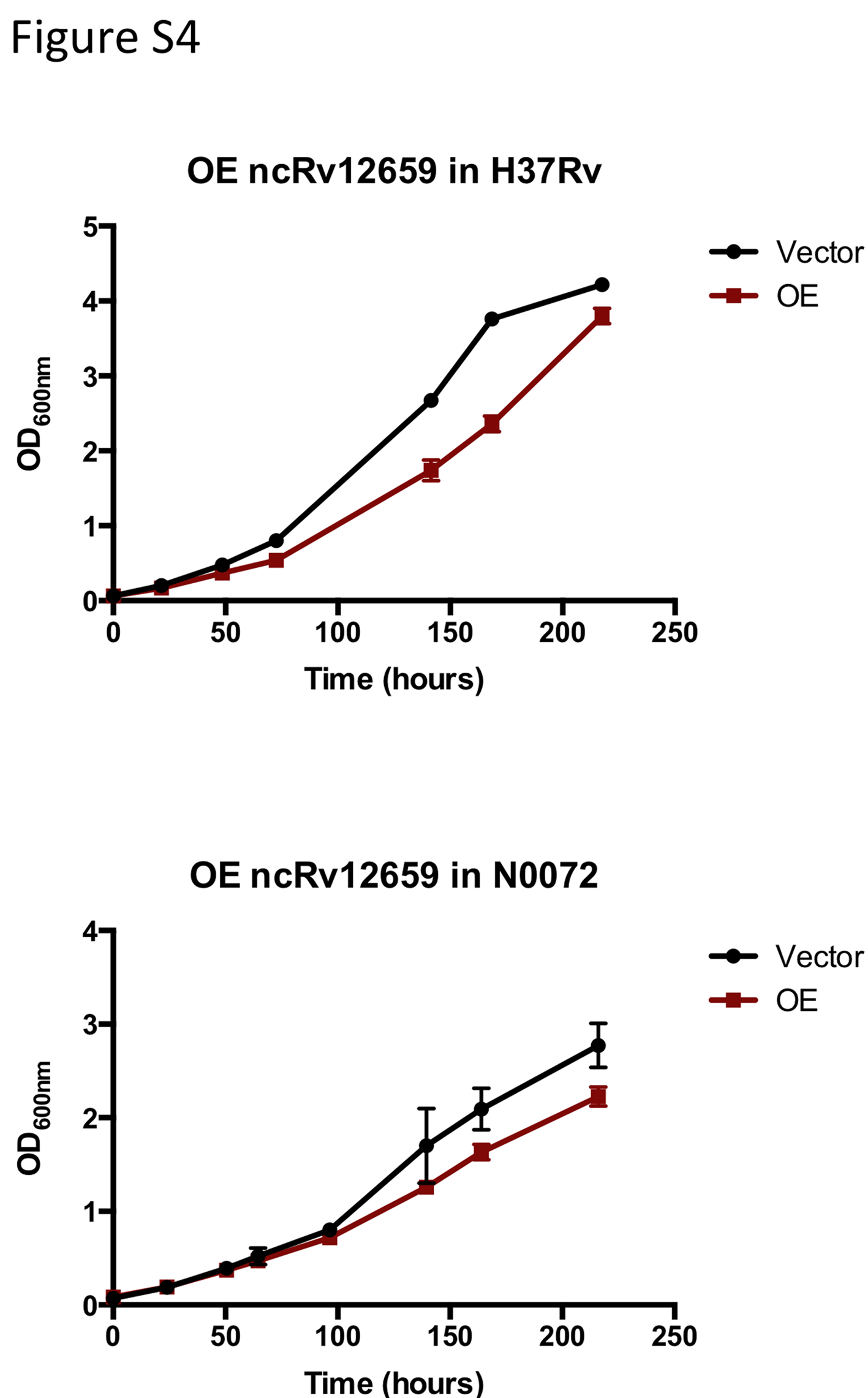

Supplement: Figure S4 — Growth of M. tuberculosis upon ncRv12659 overexpression. The curves illustrate that both strains of M. tuberculosis had a significant growth defect when expressing high amounts of ncRv12659. (TIF) [file pone.0080047.s004.tif]

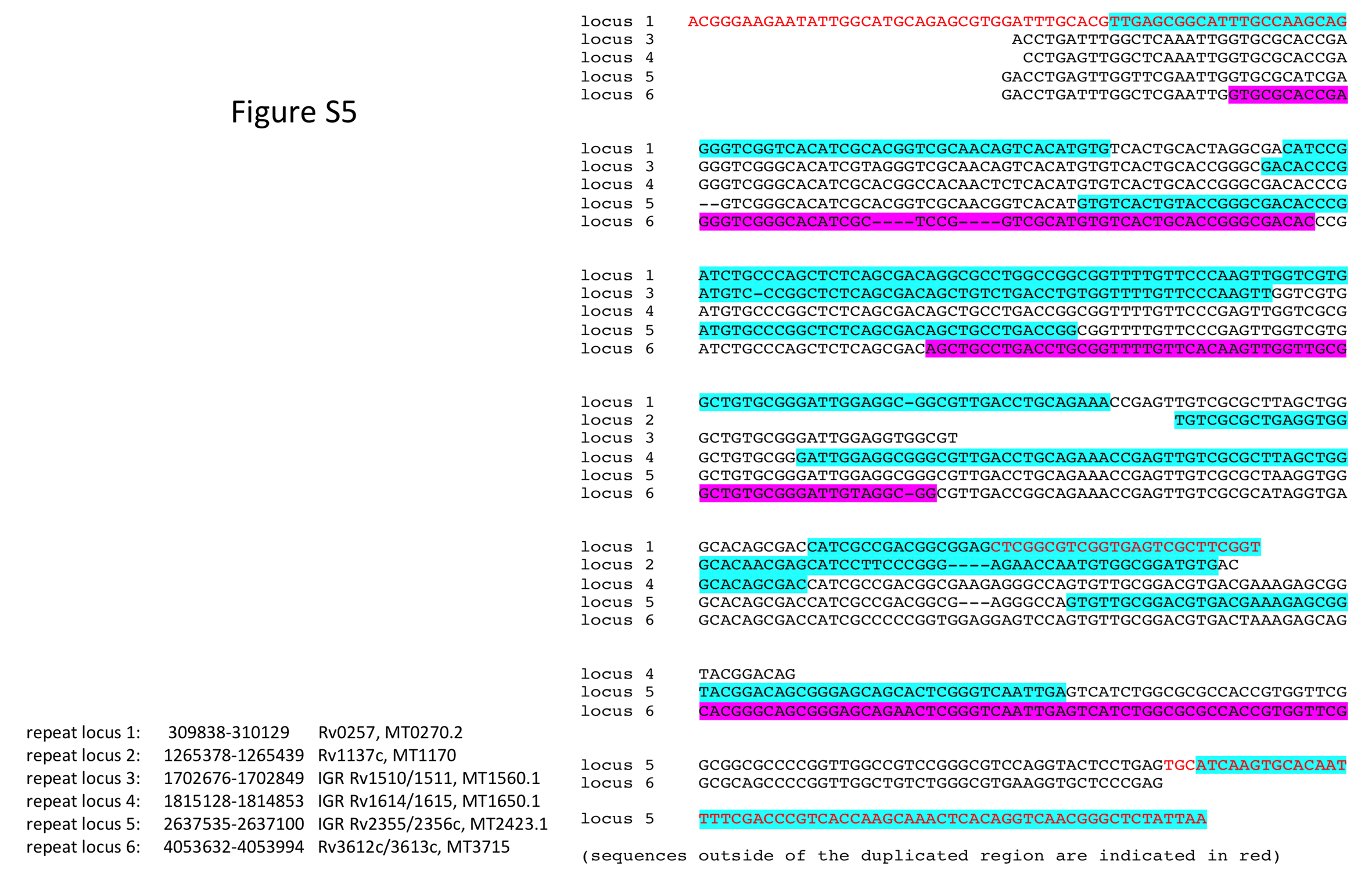

Supplement: Figure S5 — Sequences of M. tuberculosis repeat loci. Sequence alignments of the repeat loci with homology to Rv0257. The diagram illustrates the sequences of the individual repeats, their location and the genes or gene regions they are associated with. Cyan highlights probes mapping in forward orientation with respect to Rv0257 orientation and magenta highlights probes mapping in the antisense orientation. (TIF) [file pone.0080047.s005.tif]
