# Supplementary material for: A Small RNA Encoded in the Rv2660c Locus of Mycobacterium tuberculosis Is Induced during Starvation and Infection
Source: PLoS One. 2013 Dec 12;8(12):e80047. doi: 10.1371/journal.pone.0080047 (PMC3861185; doi:10.1371/journal.pone.0080047)
Supplement: Table S2 — Primers used for quantitative RT-PCR. (DOCX) [file pone.0080047.s007.docx]

**Table S2 – qRT-PCR primers used in this study**

| **Primer Name** | **Sequence (5’-3’)** |
| --- | --- |
| 16sTqmF | TCCCGGGCCTTGTACACA |
| 16sTqmR | CCACTGGCTTCGGGTGTTA |
| 5’q12659F | CACCGGCCCACTAGTGAAAC |
| 5’q12659R | GGATGAAACCGCAGGTCAA |
| 3’q12659F | TGACCTGCGGTTTCATCCA |
| 3'q12659R | TGCACCGAGTCAGTTCACATTTA |
| 2658c TqmF | CGAAGCTTCACGAGCTGAGA |
| 2658c TqmR | GCGCCTCTGCTATTGATGGA |
| 2659cTqmF | GGCTGAAGCAGCGTGGAAT |
| 2659c TqmR | TTGTCCAGCAGTTTGCGATAGT |
| Rv3229c (DesA3) TqmF | GGCGCTGCGGTTCATG |
| Rv3229c (DesA3) TqmR | CAGGTCCGGATAGAGGTGATG |
